# Supplementary material for: Urban risk factors for human Rift Valley fever virus exposure in Kenya
Source: PLOS Glob Public Health. 2022 Jul 14;2(7):e0000505. doi: 10.1371/journal.pgph.0000505 (PMC10021321; doi:10.1371/journal.pgph.0000505)
Supplement: S1 Appendix — (PDF) [file pgph.0000505.s001.pdf]

Demography

Record ID ______________________

GPS Data

House Latitude ______________________

House Longitude ______________________

Head of Household (HOH) Information

Note to Interviewer: one Demographic Questionnaire should be completed per household. Ideally, the Head of Household will complete this Questionnaire, but if that person is not available, another adult can complete it.

Will this participant be completing the Demographic Questionnaire for this household?
- ◯ Yes
- ◯ No

Are you the head of household?

Je wewe ni kiongozi wa jamii?

In e wuon ot?
- ◯ Yes
- ◯ No

If no, what is the relationship of the participant to the head of household?

Note to Interviewer: This question is asking WHAT THE PARTICIPANT IS to the head of household. Example: if the Head of Household is the father of the participant, the answer should be 'child.'
- ◯ Spouse
- ◯ Child
- ◯ Parent
- ◯ Sibling
- ◯ In-law
- ◯ Cousin
- ◯ Step-child
- ◯ Step-parent
- ◯ Other

If other, what is the relationship of the participant to the head of household? ______________________

What language do you speak?<br><br>Ni lugha gani unaongea?<br><br>Lwacho dhok mane?	☐ Buganda<br>☐ Digo<br>☐ Duruma<br>☐ English<br>☐ Kalenjin<br>☐ kamba<br>☐ Kikuyu<br>☐ Kisii<br>☐ Kuria<br>☐ Luhya<br>☐ Luo<br>☐ Maasai<br>☐ Nandi<br>☐ Nubian<br>☐ Swahili<br>☐ Other<br>☐ Refused
If "Other" what other language do you speak?<br><br>Kama ni nyingine ni lugha gani unayoiongea?<br><br>Kantie dhok moko gin mage?	________________________
What tribe are you?<br><br>Wewe ni kabila gani?<br><br>In ja dhok mane?	◯ Buganda<br>◯ Digo<br>◯ Duruma<br>◯ Giriama<br>◯ Kalenjin<br>◯ Kamba<br>◯ Kikuyu<br>◯ Kisii<br>◯ Kuria<br>◯ Luhya<br>◯ Luo<br>◯ Maasai<br>◯ Nandi<br>◯ Nubian<br>◯ Other<br>◯ Refused
If "Other" what tribe are you a part of?<br><br>Kama ni nyingine ni kabila gani?<br><br>Kantie moro, in dhok mane?	________________________
What religion do you practice?<br><br>Je, wewe ni wa dini gani?<br><br>In jadin mane?	◯ Christian<br>◯ Islam/Muslim<br>◯ No Religion<br>◯ Other

If "other," what other religion do you practice?

Kama ni nyingine ni dini gani?

Kantie moro en mane?

____

What is your marital status?

Je, uko kwa ndoa?

Chal mari mar keny en mane?

- ◯ Single
- ◯ Married
- ◯ Divorced
- ◯ Widowed
- ◯ Separated
- ◯ Other
- ◯ Refused

If "Other," what is your marital status?

Kama ni nyingine ndoa yako ni ya aina gani?

Kantie moro chal mari mar keny en mane?

____

What is the gender of the head of household?

Ni jinsia gani ya kiongozi wa jamii?

Wuon ot en miyo koso dichuo?

- ◯ Male
- ◯ Female

What is the age of the head of household, in years?

Kiongozi wa jamii ako na umri wa miaka ngapi?

Wuon ot en jahigni adi?

____

What languages does the head of household speak?

Kiongozi wa jamii anaongea lugha gani?

Wuon ot wacho dhok mage?

- ☐ Buganda
- ☐ Digo
- ☐ Duruma
- ☐ English
- ☐ Kalenjin
- ☐ Kamba
- ☐ Kikuyu
- ☐ Kisii
- ☐ Kuria
- ☐ Luhya
- ☐ Luo
- ☐ Maasai
- ☐ Nandi
- ☐ Nubian
- ☐ Swahili
- ☐ Other
- ☐ Refused

If "Other", what other language does the head of household speak? __________________________________

Kama ni nyingine kiongozi wa jamii anaongea lugha gani?

Kantie dhok moko ma wuon ot wacho gin mage?

What tribe is the head of household?

Kiongozi wa jamii ni wa kabila gani?

Wuon ot en jadhok mane?

- ◯ Buganda
- ◯ Digo
- ◯ Duruma
- ◯ Giriama
- ◯ Kalenjin
- ◯ Kamba
- ◯ Kikuyu
- ◯ Kisii
- ◯ Kuria
- ◯ Luhya
- ◯ Luo
- ◯ Maasai
- ◯ Nandi
- ◯ Nubian
- ◯ Other
- ◯ Refused

If "Other", what tribe is the head of household a part of? __________________________________

Kama ni nyingine kiongozi wa jamii ni wa kabila gani?

Kantie moro wuon dala jadhok mane?

What religion does the head of household practice?

Kiongozi wa jamii ni wa dini gani?

Wuon dala jadin mane?

- ◯ Christian
- ◯ Islam/Muslim
- ◯ No Religion
- ◯ Other

If "Other", what other religion does the head of compound practice? __________________________________

Kama ni nyingine kiongozi wa jamii ni wa dini gani?

Kantie dini moro,wuon dala jading mane?

What is the marital status of the head of household?

Je, kiongozi wa jamii ako kwa ndoa?

Chal mari mar keny en mane?

- ◯ Single
- ◯ Married
- ◯ Divorced
- ◯ Widowed
- ◯ Separated
- ◯ Other
- ◯ Refused

If "Other", what is the marital status of the head of household? __________________________________

Kama ni nyingine kiongozi wa jamii ako kwa ndoa gani?

Kantie moro chal mar keny mar wuon dala en mane?

Household Characteristics

How many adults sleep in this household? __________________________________

Je, ni watu wazima wangapi wanalala kwa hi nyumba?

Gin jii adi madongo manindo eodni?

How many children sleep in this household? __________________________________

Je, ni watoto wangapi wanalala kwa hi nyumba?

Nyithindo adi manindo ei odni?

How many rooms in this household are used for sleeping? __________________________________

Watu hulala kwa vyumba ngapi kwa hii nyumba?

Nitie kidienje adi mag nidno e odni?

How long has your family lived in this house?

Jamii yako imeishi kwa hii nyumba kwa mda ga?

Familia ni osedak kaa kuom kinde maron nade?

- ◯ Less than 6 months
- ◯ Between 6 months and 12 months
- ◯ 1 - 5 years
- ◯ 6 - 10 years
- ◯ More than 10 years

What is the main source of drinking water for this household?

Ni chanzo gani muhimu cha maji ya kunywa kwa hii jamii?

Ugolo pii modho kanye?

- ◯ Piped into yard/plot
- ◯ Public tap/standpipe
- ◯ Tube Well or Borehole
- ◯ Protected Well
- ◯ Unprotected Well
- ◯ Protected Spring
- ◯ Unprotected Spring
- ◯ Rainwater
- ◯ Tanker truck
- ◯ Cart with small water tank
- ◯ Surface water (River/Dam/Lake/Pond/Stream/Canal/Irrigation Channel)
- ◯ Bottled Water
- ◯ Other

If other, please specify your main source of drinking water<br><br>Kama nyingine, eleza ni chanzo gani muhimu cha maji ya kunywa kwa hii jamii?<br><br>Kantie moro en mane ma utiyo go kaka pii modho?	__________________________
Where is this water source located?<br><br>Chanzo muhimu cha maji kiki wapi?<br><br>Kuma uyude pii modhoni ni kanye?	◯ In own house<br>◯ Outside own house, but in own building or compound<br>◯ Outside own building/compound
If outside own dwelling, how many minutes does it take to go there, get water, and come back to your house?<br><br>Unachukua dakika ngapi kuenda kuteka maji,na kurudi kwa nyumba?<br><br>Ikawo dakika adi kidhi kuma iome pii dhii gi duogo?<br><br>INSTRUCTIONS FOR DATA COLLECTOR: Write in a number, or else type in 999 if the answer is "I don't know"	__________________________<br>(Please enter 999 if the respondent doesn't know how long it takes to get water)
What kind of toilet facility do members of your household primarily use?<br><br>Ni choo ya aina gani jamii yako wanatumia mara nyingi?<br><br>Uhinyo tiyo gi choo machal nade?	◯ Flush to sewer system<br>◯ Flush to septic tank<br>◯ Flush to pit latrine<br>◯ Flush to somewhere else, or don't know where<br>◯ Pit latrine, ventilated and improved (Improved pit with a ventilation pipe)<br>◯ Pit latrine with slab (pit with any cleanable material - concrete, wood, porcelain, etc - feet rest on this material)<br>◯ Pit latrine without slab (open pit without any materials, feet rest on bare ground)<br>◯ Composting toilet (toilet seat attached to a removable container that collects the waste)<br>◯ Bucket toilet (container that is used and then emptied)<br>◯ No Facility/bush/field<br>◯ Other
If other, what kind of toilet facility does your household primarily use?<br><br>Kama nyingine, ni choo ya aina gani jamii yako wanatumia mara nyingi?<br><br>Kantie moro to en choo machal nade ma joodni tiyo go hinyne?	__________________________

Do you share this toilet facility with other households?<br><br>Je, munatumia choo na jamii zingine?<br><br>Bende utiyo gi choo kog ute mamoko?	◯ Yes<br>◯ No<br>◯ Don't Know
If yes, how many other households use this facility?<br><br>Kama ndio, jamii zingine ngapi hutumia hii choo?<br><br>To kantie gin ute adi matiyogi choono?	◯ More than 10<br>◯ Less than 10<br>◯ Don't Know
Are there objects around your home that collect water?	◯ Yes<br>◯ No<br>◯ Don't know
If yes, what kinds of objects collect water?	☐ Animal feeding troughs<br>☐ Bins<br>☐ Buckets<br>☐ Coconut shells<br>☐ Fallen leaves<br>☐ Jars<br>☐ Jerry cans<br>☐ Larger plastic containers (ex: discarded sinks or buckets)<br>☐ Plastic bags<br>☐ Plastic bottles<br>☐ Pots<br>☐ Street drains or gutters<br>☐ Tanks<br>☐ Tins/cans<br>☐ Tires<br>☐ Treeholes<br>☐ Water storage containers (ex: drums)<br>☐ Wells<br>☐ Other<br>☐ Don't know
If another type of object collects water, what kind?	__________________________________

Which of the following does your household have?

Ni gani kwa hivi vitu vifutavyo muko navyo kama jamii?

Kuom gigi en mane ma joodi nigo?

	Yes	No
A bicycle	◯	◯

A car or truck	◯	◯
A domestic worker	◯	◯
Electricity	◯	◯
A microwave oven	◯	◯
A mobile telephone	◯	◯
A motorcycle or motor scooter	◯	◯
A radio	◯	◯
A refrigerator	◯	◯
A sofa	◯	◯
A television	◯	◯

Does anyone in your household cook for the household?

Kunayo yeyote nyumbani kwenu ambaye hupikia watu nyumbani?

Bende nitie ng’ama tedo ne jii eodu?

◯ Yes
◯ No

What type of fuel does your household primarily use for cooking?

Ni nini munatumia mara nyingi kwa kupika?

Utiyo kod ang’o e tedo hinyne?

◯ Biogas (fuel from organic material such as animal manure or vegetable matter)
◯ Charcoal
◯ Electricity
◯ "Gas"/LPG (liquid petroleum gas))
◯ Paraffin/kerosene
◯ Solar
◯ Straw/shrubs/grass
◯ Wood
◯ No food cooked in household
◯ Other

If "Other" what type of cooking fuel does your household primarily use?

Kama ni nyingine, munatumia nini mara nyingi kwa kupika?

Kantie moro utiyo kod ang’o e tedo hinyne?

____

Where is your household's cooking usually done?

Mara nyingi huwa munapikia wapi?

Utedo ga kanye hinyne?

☐ In the house
☐ In a separate building
☐ Outdoors
☐ No food cooked by household members
☐ Other

If "other", where is the cooking usually done?
Kama ni kwingine, mara nyingi huwa munapikia wapi?
Kantie moro kara utedo ga kanye hinyne?

____

Record Observation: Main material of the floor	◯ Carpet<br>◯ Cement<br>◯ Ceramic Tiles<br>◯ Dung<br>◯ Earth/Sand<br>◯ Palm/Bamboo<br>◯ Parquet or polished wood<br>◯ Vinyl or asphalt strips<br>◯ Wood plank<br>◯ Other
If "Other" what is the main material of the floor?	__________________________________
Record observation: Main material of roof	◯ Thatch/Grass/Makuti<br>◯ Dung/Mud/Sod<br>◯ Iron sheets<br>◯ Tin cans<br>◯ Asbestos<br>◯ Concrete<br>◯ Roof tiles<br>◯ Other
If "Other" what is the main material of the roof?	__________________________________
How many windows does the household have?<br><br>Nyumba iko na madirisha ngapi?<br><br>Ot nigi dirisni adi?	__________________________________
Are there screens on the windows to the outside?<br><br>Je, kuna waya ya kuzuia mbu kwa madirisha?<br><br>Bende nitie waya mageng'o suna e dirisa?	◯ No<br>◯ Yes, but only some of the windows have screens<br>◯ Yes, all the windows have screens
Does your family own this house in which you live?<br><br>Je familia yako wanamiliki nyumba hii mnayoishi?<br><br>Bende joodu e we got mudake ni?	◯ Yes<br>◯ No<br>◯ Refused
Through what arrangement does your family live here?<br><br>Je wanaoishi hapa wana mpangilio ipi?<br><br>Ere kaka udak eodu kaa?	◯ Pays rent/lease<br>◯ No rent, with consent of owner<br>◯ No rent, squatting<br>◯ Refused

Does your family own the land on which this structure (house, flat, shack) sits?<br><br>Je, hii shamba penye mumejenga ni lenu?<br><br>Kama ugedeni lopu?	◯ Yes<br>◯ No<br>◯ Refused
Through what arrangements does your family live on this land?<br><br>Je wanaoishi hapa wana mpangilio ipi?<br><br>Ere kaka udak eodu kaa?	◯ Pays rent/lease<br>◯ No rent, with consent of the owner<br>◯ No rent, squatting<br>◯ Refused
Does any member of your household own any agricultural land, anywhere?<br><br>Je, kuna yeyote kwa jamii yenu anamiliki shamba la kulima?<br><br>Bende nitie ng'at moro amora kuomu man gipuodho?	◯ Yes<br>◯ No
If yes, approximately how many acres are owned?<br><br>Kwa kipimo mna ekari ngapi?<br><br>Kipimo un gi heka adi?<br><br>INSTRUCTIONS FOR DATA COLLECTOR: Write in a number, or else type in 999 if the answer is "I don't know"	__________________________________<br>(Please enter 999 if the respondent doesn't know how long it takes to get water)
Does this household own any livestock, or poultry?<br><br>Je, kuna yeyote kwa jamii yenu anamiliki wanyama wa kufuga?<br><br>Bende nitie ng'at moro amora kuomu majapith lee mag dala?	◯ Yes<br>◯ No
If yes, check all that apply, and give an approximate number	☐ Goats<br>☐ Sheep<br>☐ Chickens<br>☐ Ducks<br>☐ Camels<br>☐ Horses<br>☐ Donkeys<br>☐ Pigs<br>☐ Cattle<br>☐ Other

If "Other" what kind of livestock do you keep? __________________________________

Kama nyingine, ni mfugo wa aina gani?

Kantie moko gin kaka pith mage?

Number of goats __________________________________

Number of sheep __________________________________

Number of chickens __________________________________

Number of ducks __________________________________

Number of camels __________________________________

Number of horses __________________________________

Number of donkeys __________________________________

Number of pigs __________________________________

Number of cattle __________________________________

Number of other livestock __________________________________

Notes:

If you would like to note anything about the interview or household, please put these observations here ____

Healthy Visit Survey

Record ID ______________________

Note to interviewer: If the 'Visit Screening' form recommended that this participant have a Sick Visit conducted (they currently have a fever or have a history of fever in the past 3 days), STOP! Do not complete this questionnaire. Instead, a Clinical Officer should come and conduct a Sick Visit with this participant.

Occupation

Are you currently in school?

Je,ungali shuleni?

Gi esechegi bende pod idhi school?

◯ Yes
◯ No

What is the name of your school?

Shule yenu yaitwaje?

____

If yes: Is your school in Kisumu?

Shule yako ingali Kisumu?

Sikundu yudore Kisumu?

◯ Yes
◯ No

If yes: Is your school in Ukunda?

Shule yako ingali ukunda?

Sikundi yudore ukunda?

◯ Yes
◯ No

If no, where is your school?

Je, shule yako iko wapi?

Sikundi ni kanye?

INSTRUCTIONS FOR DATA COLLECTOR: Try to get as close to the exact location as possible by asking whatever would be needed for you to pinpoint the location on a map. For instance, if the participant gives the name of a town and you know the place, you can stop there. If they cannot give a town name, try to get the name of the area or region.

Neighborhood/estate ______________________

City/town ______________________

County ______________________

Region	__________________________________
Country (other than Kenya)	__________________________________
Do you board (sleep) there?<br><br>Je wewe hulala humo?<br><br>Inindo ga kuro?	◯ Yes<br>◯ No
Do you have a job or do any work to earn money?<br><br>Gani ndio kazi yako muhimu?<br><br>En tich mane mitimo kaka yuto mari maduong?	◯ Yes<br>◯ No
Do you have a job or do any work to earn money?<br><br>Gani ndio kazi yako muhimu?<br><br>En tich mane mitimo kaka yuto mari maduong?	◯ Agricultural worker or farmer<br>◯ Artisan or craftsman<br>◯ Business Owner<br>◯ Butcher or slaughterhouse worker<br>◯ Clerical<br>◯ Cook<br>◯ Domestic service<br>◯ Driver<br>◯ Electrician<br>◯ Fisherman<br>◯ Gardener<br>◯ Government Worker<br>◯ Guard<br>◯ Healthcare worker<br>◯ Home Caregiver<br>◯ Hotel room keeping/service<br>◯ Manual Laborer<br>◯ Merchant or trader<br>◯ Military<br>◯ Office worker<br>◯ Police<br>◯ Researcher<br>◯ Salon worker<br>◯ Sells goods in a market or small shop<br>◯ Student<br>◯ Teacher<br>◯ Technical or managerial<br>◯ Tourism<br>◯ Traditional Healer<br>◯ Veterinarian<br>◯ Other

If "other" what is your primary job?

Kama nyingine, gani ndio kazi yako muhimu?

Kantie moro itiyo tich mane mar yuto ni pile kapile?

____

Do you have a second type of work to earn money?

Gani ndio kazi yako muhimu?

En tich mane mitimo kopogore gi mitimo ga pile?

- ◯ Yes
- ◯ No

Do you have a second type of work to earn money?

Gani ndio kazi yako muhimu?

En tich mane mitimo kopogore gi mitimo ga pile?

- ◯ Agricultural worker or farmer
- ◯ Artisan or craftsman
- ◯ Business Owner
- ◯ Butcher or slaughterhouse worker
- ◯ Clerical
- ◯ Cook
- ◯ Domestic service
- ◯ Driver
- ◯ Electrician
- ◯ Fisherman
- ◯ Gardener
- ◯ Government Worker
- ◯ Guard
- ◯ Healthcare worker
- ◯ Home Caregiver
- ◯ Hotel room keeping/service
- ◯ Manual Laborer
- ◯ Merchant or trader
- ◯ Military
- ◯ Office worker
- ◯ Police
- ◯ Researcher
- ◯ Salon worker
- ◯ Sells goods in a market or small shop
- ◯ Student
- ◯ Teacher
- ◯ Technical or managerial
- ◯ Tourism
- ◯ Traditional Healer
- ◯ Veterinarian
- ◯ Other

If "other" what is your secondary occupation?

Kama nyingine, gani ndio kazi yako ya pili?

Kantie moro tich mane mitimo ga tenge?

____

Education

What is your highest level of education?

Je, masomo yako ya juu ni gani?

Sombi mamalo en mane?

- ◯ No education
- ◯ Some primary school
- ◯ Completed primary school
- ◯ Some secondary school
- ◯ Completed secondary school
- ◯ College/university
- ◯ Technical college
- ◯ Other

If other, what is your highest level of education?

____

If under 18 years old:

What is your mother's highest level of education?

Kama uko chini ya miaka 18, je mamako mzazi alifika wapi kwa masoma?

Kain e bwo higa apar ga abor, somo mamalo mar mamani en mane?

- ◯ No education
- ◯ Some primary school
- ◯ Completed primary school
- ◯ Some secondary school
- ◯ Completed secondary school
- ◯ College/university
- ◯ Technical college
- ◯ Don't know
- ◯ Other

If other, what is your mother's highest level of education?

____

Dengue and Chikungunya Knowledge Questions

Before you were introduced to this study, had you heard of dengue?

Kabla ya leo, je uliwai sikia kuhusu dengue?

Kaopogre gi kawuono bende isaga winjo tuo mar dengue?

- ◯ Yes
- ◯ No

If yes, where did you hear about it?

Kama ndio, ulisikia wapi?

Kaisewinjo, niwinje kanye?

- ☐ Family member or relative
- ☐ Friend
- ☐ Neighbor
- ☐ Healthcare worker
- ☐ Sign somewhere
- ☐ Newspaper
- ☐ Radio
- ☐ Another research project
- ☐ Other

Do you do anything to prevent getting dengue?

Kama ndio, je unafanya chochote kuuzuia?

Kantie,bende nitie gima itime mar geng’e?

- ◯ Yes
- ◯ No

If yes, what do you do to prevent dengue?

Kama ndio, nini?

Kanitie en ang'o?

____

Before you were introduced to this study, had you heard of chikungunya?

Kabla ya leo, je uliwai sikia kuhusu chikungunya?

Kopogore gi kawuono bende isegawinjo chikungunya?

◯ Yes
◯ No

If yes, where did you hear about it?

Kama ndio, ulisikia wapi?

Kaisewinjo, niwinje kanye?

☐ Family member or relative
☐ Friend
☐ Neighbor
☐ Healthcare worker
☐ Sign somewhere
☐ Newspaper
☐ Radio
☐ Another research project
☐ Other

Do you do anything to prevent getting chikungunya?

Kama ndio, je unafanya chochote kuuzuia?

Kantie,bende nitie gima itime mar geng'e?

◯ Yes
◯ No

If yes, what do you do to prevent chikungunya?

Kama ndio, nini?

Kanitie en ang'o?

____

Moved

During the last 6 months, have you spent time living and sleeping outside your home?

Kwa muda wa miezi 6 iliopita, je umewai ishi na kulala mbali na boma lenu?

Kuom dweche auchie (6) mosekadho,bende isedak kata nindo oko mabor gi dalani?

◯ Yes
◯ No

If yes, where did you stay?

Kama ndio, uliishi wapi?

Ka isedak en kanye?

INSTRUCTIONS FOR DATA COLLECTOR: Try to get as close to the exact location as possible by asking whatever would be needed for you to pinpoint the location on a map. For instance, if the participant gives the name of a town and you know the place, you can stop there. If they cannot give a town name, try to get the name of the area or region.

Neighborhood/estate	__________________
City/town	__________________
County/region	__________________
Country (if other than Kenya)	__________________
If yes, how long did you stay there?<br><br>Kama ndio, ulishi huko kwa muda gani?<br><br>Ka isedak,isebet kuro kuom ndalo marom nade?	◯ Less than 1 week<br>◯ 1 week - 1 month<br>◯ 1 month - 3 months<br>◯ 3-6 months<br>◯ Other
If "other," how long did you stay there?<br><br>Note to interviewer: Please feel free to add any comments regarding the participant's movements here	______________________
Is there a second place you lived and slept during the last 6 months?<br><br>Kuna pahali pa pili ulishi na kulala kwa muda wa miezi 6 iliopita?<br><br>Bende nitiere kamoro ma ariyo misedak kata nindo oko mabor gi dalani?	◯ Yes<br>◯ No

If yes, where did you stay?

INSTRUCTIONS FOR DATA COLLECTOR: As above, try to get as close to the exact location as possible.

Neighborhood/estate	__________________
City/town	__________________
County/Region	__________________
Country (if other than Kenya)	__________________
If yes, how long did you stay there?	◯ Less than 1 week<br>◯ 1 week - 1 month<br>◯ 1 month - 3 months<br>◯ 3-6 months<br>◯ Other

If "other," how long did you stay there?

Note to interviewer: Please feel free to add any comments regarding the participant's movements here

____

Mosquito Exposure

Does your household have bednets?

Je jamii yako muko na neti ya mbu?

Bende odi nitie gi net mar suna?

◯ Yes
◯ No

How many bednets do you have?

Je, muko na neti ngapi za mbu?

Un gi nede adi mag suna?

____

Did you sleep under a mosquito net last night?

Je, ulilala ndani ya neti ya mbu usiku uliopita?

Bende ni nindo ei net mar suna nyoro gotieno?

◯ Yes
◯ No

How often do you sleep under a mosquito net?

Ni mara ngapi unalala ndani ya neti ya mbu?

Inindo ga e buo net kuom kinde marom nade?

◯ Always
◯ Sometimes
◯ Rarely
◯ Never

When is the last time your house got a new mosquito net?

Ni lini mara ya mwisho mulipata neti mpya ya mbu?

Nuyodo net manyien kar ang’o?

◯ Less than 1 month ago
◯ 1-6 months ago
◯ 7-12 months ago
◯ 1-5 years ago
◯ more than 5 years ago

When you got your nets, were any of them treated with insecticides to repel or kill mosquitoes before you bought them?<br><br>Ulipopata neti zako,kulikuwa na ziliyotibiwa na dawa ya kuzuia wadudu ama kuua mbu kabla ya kuzinunua?<br><br>Kaniyudo net,bende nenitie moro kuomgi mane othiethi gi yath mar geng’o suna kata negogi kapok ninyiewogi?	◯ Yes<br>◯ No<br>◯ Don't know
Have any of your bednets been treated with insecticides after they were purchased?<br><br>Je, kuna neti ya mbu yeyote imewai wekwa dawa ya wadudu baada ya kununuliwa?<br><br>Nyaka nunyiew nede gi bende nite mosethiedhi gi yath kute?	◯ Yes<br>◯ No<br>◯ Don't know
If yes, how many months ago were they treated with insecticide?<br><br>Kwa mda wa miezi mingapi zilizopita zilitibiwa na dawa ya kuzuia wadudu?<br><br>Kuom kinde marom nade mane ithiethо gi gi yath geng’o suna?<br><br>INSTRUCTIONS FOR DATA COLLECTOR: Write in a number, or else type in 999 if the answer is "I don't know"	__________________________________<br>(Please enter 999 if the respondent doesn't know how long it takes to get water)
In the past two weeks, have you been bitten by mosquitoes?<br><br>Kwa muda wa juma mbili zilizopita, je umewai umwa na mbu?<br><br>Ei kinde mar jumbe ariyo mokadho bende suna osekayi?	◯ Yes<br>◯ No<br>◯ Don't Know
If yes, when did the mosquitoes bother you?<br><br>Kama ndio, zilikuuma wakati gani?<br><br>To ka osekayi nokayi seche mage?	☐ Day<br>☐ Night<br>☐ Don't know

Approximately how often are you bitten by mosquitoes?

Kwa kadri, unaumwa na mbu mara ngapi?

Kinyalo pimo suna kayi ga marom nade?

- ◯ Daily
- ◯ Weekly
- ◯ Monthly
- ◯ Yearly
- ◯ Don't Know

Mosquito Control

In the past two weeks...

Kwa juma mbili zilizopita...

Kuom jumbe ariyo mokadho...

	Yes	No	Don't Know
Have you used mosquito repellent? / Je, umejipaka mafuta ya kufukuza mbu? / Bende isewirori gi moo mariembo suna?	◯	◯	◯
Have you used mosquito coils in/around your house? / Je, umetumia dawa ya kuchoma ya kufukuza mbu ndani au karibu na nyumba yako? / Bende isetiyo gi yath miwang'o mar riembo suna ei ot kata aluora mara ot?	◯	◯	◯
Have you used insecticide in/around your house? / Je, umetumia dawa ya wadudu ndani au karibu na nyumba yako? / Bende isetiyo gi yath kute iot kata aluora mar ot?	◯	◯	◯
Has anyone come into your house to spray the interior walls against mosquitoes? / Je, kuna mtu yeyote aliyepuliza dawa ya mbu kwa kuta za ndani ya nyumba yako? / Bende ng'ato ang'ata osebiro go yath suna e kor ot mar ii odi?	◯	◯	◯

Latest Fever Questions

Have you had a fever in the last six months?

Je, umekuwa na joto jingi mwilini kwa muda wa miezi 6 iliopita?

Bende isebet gi liet mar del kuom kinde mar dweche 6 mokadho?

◯ Yes
◯ No

If yes, how long did the symptoms for your latest fever last?

Kama ndio, hizi dalili zilikuwa kwa muda gani?

Ka isebet go, ranyisi gi nibet go kuom kinde kata ndalo marom nade?

FOR THE INTERVIEWER: Please enter how long the participant's fever lasted. Not all fields need to be filled out. For example, if the participant says "2 days," just put "2" in the 'days' field and leave the other fields blank. If "3 months," put "3" in the 'months' field

Days ________________________

Weeks ________________________

Months ________________________

If yes, did you seek medical care for your most recent fever?

Kama ndio, ulitafuta huduma ya matibabu kwa joto jingi mwilini uliokuwa nayo hivi karibuni?

Kaisebet go,bende nimanyo yor thieth ne liet mar del manyocha in godo machiegni ni?

◯ Yes
◯ No
◯ Don't Know

If yes, what was the source of this care?

Kama ndio, ulipata wapi haya matibu?

To kane imanyo, niyudo thieth kanye?

☐ Clinic or hospital
☐ Community health worker outside a clinic or hospital
☐ Pharmacy or chemist
☐ Care from a family member or acquaintance NOT trained as healthcare worker
☐ Traditional healer
☐ Other

If other, what other type of medical care did you receive? ________________________

If a clinic or hospital, which clinic or hospital did you go to?<br><br>Ulienda kwa hospitali gani?<br><br>Nidhi osiptal mane?	☐ Obama Childrens Hospital<br>☐ Likoni Health Center<br>☐ Jaramogi Odinga Referral Hospital<br>☐ Ukunda Medical Center<br>☐ Kisumu Hospital<br>☐ Msambweni County Referral Hospital<br>☐ Migosi Subcounty Referral Hospital<br>☐ Kwale Subcounty Hospital<br>☐ Other
If other, specify where the participant sought medical care.	__________________________________
Did you see a Clinical Officer FROM THIS STUDY while you were sick?<br><br>Kama ndio, afisa wa matibabu wa utafiti huu alikuja kukuona ulipokuwa na joto jingi mwilini?<br><br>To kanitie, bende laktar mochung ne thieth mar nonroni noneni kane in gi liet mar del?	◯ Yes<br>◯ No
If yes, where did you see the clinical officer?<br><br>Kama ndio, mulionana wapi na afisa wa matibabu?<br><br>To ka ee,nineno laktar mochung ne thieth kanye?	◯ At home<br>◯ At healthcare center<br>◯ Other
If "Other" where did you see the clinical officer?	__________________________________

Symptoms during most recent fever
Wakati ulikuwa na joto jingi mwilini hivi karibuni, ni ishara gani zingine ulikuwa nazo?
E seche mane in gi liet mar del nyocha machigni, ranyisi mane kendo mane in go?

Instructions for clinical officer: ask the participant about EACH specific organ system listed below. If they say they had a problem with that organ system, check the box and then you will be prompted to specify exactly what the problem was.

	Yes	No	Unsure/Don't Know
PROBLEMS WITH THE HEAD, BRAIN, or NECK / Shida za kichwa, ubongo ama shingo / In gi rem mar wich,obuongo kata ng'uti	◯	◯	◯
Confusion	◯	◯	◯
Dizziness	◯	◯	◯

Headache	◯	◯	◯
Neck swelling	◯	◯	◯
Seizures	◯	◯	◯
Stiff neck	◯	◯	◯
PROBLEMS WITH EYES, EARS, NOSE, or THROAT / Shida za macho, maskia, mapua ama koo / In gi rem mar wang'I it,umi kata duondi	◯	◯	◯
Eye discharge	◯	◯	◯
Eye pain	◯	◯	◯
Eye redness	◯	◯	◯
Yellow eyes	◯	◯	◯
Ear discharge	◯	◯	◯
Ear pain	◯	◯	◯
Runny nose	◯	◯	◯
Sore throat	◯	◯	◯
PROBLEMS WITH CHEST OR BREATHING / Shida za kupumua ama kifua / In gi rem mar yueyo kata kori	◯	◯	◯
Chest Pain	◯	◯	◯
Cough	◯	◯	◯
Difficulty breathing	◯	◯	◯
STOMACH PROBLEMS / Shida za tumbo / In gi ich maremo	◯	◯	◯
Abdominal Pain	◯	◯	◯
Constipation	◯	◯	◯
Diarrhea	◯	◯	◯
Nausea or Vomiting	◯	◯	◯
PROBLEMS WITH MUSCLES, JOINTS, OR LIMBS / Shida za kuuma na misuli ama viungo / In gi rem mar ondhundho kata fuondni ni	◯	◯	◯
Joint pain	◯	◯	◯
Joint stiffness	◯	◯	◯
Joint swelling	◯	◯	◯
Muscle pain	◯	◯	◯
Numbness in part of the body	◯	◯	◯
Weakness in part of the body	◯	◯	◯

PROBLEMS WITH SKIN OR BLOOD / Shida za ngozi ama damu / Rem mar pien del gi remo	◯	◯	◯
Abnormal bleeding	◯	◯	◯
Itching	◯	◯	◯
Rashes	◯	◯	◯
Sores	◯	◯	◯
Other	◯	◯	◯

During your most recent illness with fever, which other symptoms did you experience?

____

Did you receive a diagnosis for your illness?

◯ Yes
◯ No

If you received a diagnosis for your illness, what was the diagnosis?

☐ Unclear diagnosis
☐ Anemia
☐ Bacterial infection
☐ Chikungunya
☐ Dengue
☐ Ear infection
☐ Eye infection
☐ Gastritis
☐ Gastroenteritis
☐ HIV/AIDS
☐ Intestinal parasite(s)
☐ Malaria
☐ Meningitis
☐ Peptic ulcer
☐ Pneumonia
☐ Schistosomiasis
☐ Sickle cell crisis
☐ Skin infection
☐ Tonsilitis/Pharyngitis
☐ Tuberculosis
☐ Typhoid
☐ Upper respiratory tract infection ('cold')
☐ Lower respiratory tract infection ('pneumonia')
☐ Urinary Tract Infection
☐ Other

If other, what other diagnosis did the participant receive for their illness?

____

General Health

Have you been diagnosed with any medical conditions?

◯ Yes
◯ No

If yes, which ones?	☐ None<br>☐ Asthma<br>☐ Cancer<br>☐ Cerebral palsy<br>☐ Diabetes<br>☐ Epilepsy<br>☐ Heart disease<br>☐ HIV<br>☐ Hypertension<br>☐ Mental illness<br>☐ Peptic ulcer<br>☐ Sickle cell disease<br>☐ Stroke<br>☐ Tuberculosis<br>☐ Other<br>☐ Refuse
Which other medical conditions have you been diagnosed with?	__________________________________
For children under 5, was the child full term or preterm?<br><br>Kwa watoto wa umri chni ya miaka 5: huyu mtoto alizaliwa kwa wakati wake au kabla ya wakati wake?<br><br>Ne nyithindo mane ebuo higa 5: bende nyathi nonyuol kandalo ne oromo koso kapodi?	◯ Full term<br>◯ Preterm<br>◯ Do not know
For children under 5, was the child breast fed as a child?<br><br>Kwa watoto wa umri chni ya miaka 5: huyu mtoto alinyonyeshwa?<br><br>Ne nyithindo mantie e buo higni 5: bende nyathino nodhodhi?	◯ Yes<br>◯ No<br>◯ Don't Know
If yes, how many months was the participant breast fed?<br><br>Kama ndio, huyu mtoto alinyonyeshwa kwa miezi ngapi?<br><br>Kane odhoth nodhodhe kuom dweche marom nade?	◯ 1 - 6 month<br>◯ 7 - 12 months<br>◯ 13 - 24 months<br>◯ Greater than 24 months<br>◯ Do not know
Are you currently pregnant?<br><br>Je, kwa sasa we ni mja msito?<br><br>Gi seche gi iyach?	◯ Yes<br>◯ No

About how many months pregnant are you?

____

Kama ndio, takriban miezi mingapi ya uja usito?

Ka iyach, en dweche adi kama?

First Healthy Follow Up Survey

Record ID	__________________________________
Note to interviewer: This is the survey that will be filled in during the first healthy cohort follow up.	
Site	◯ Ukunda<br>◯ Kisumu
Interviewer name	◯ Said Lipi<br>◯ Charles Ng'ang'a<br>◯ Christine Njoroge<br>◯ Jael Sagina<br>◯ Lucy Njoka<br>◯ Zainab Jembe<br>◯ Godana Omar<br>◯ Paul Sillah<br>◯ Gladys Agola<br>◯ Other
If your name is not on the list above, please write it in here:	__________________________________
Interviewer name	◯ Samwel Ndire<br>◯ Stella Orwa<br>◯ Chrisphin Otieno<br>◯ Christabel Winter<br>◯ Karren Nyumbile<br>◯ Kevin Onyango<br>◯ Victoria Okuta<br>◯ Laura Wanjala<br>◯ Joel Omari<br>◯ Gladys Agola<br>◯ Charles Ronga<br>◯ Pauline Awandu<br>◯ Other
If your name is not on the list above, please write it in here:	__________________________________
Date of Interview	__________________________________
What number of visit is this?	◯ First Follow Up<br>◯ Second Follow Up<br>◯ Third Follow Up
Participant ID	__________________________________
Participant First Name	__________________________________
Participant Second name	__________________________________

Participant third name	__________________________________
Is the Name different from the one given during enrollment?	◯ Yes<br>◯ No
Gender	◯ Male<br>◯ Female
Participant Date of Birth	__________________________________
Is the Date of Birth different from the one given during enrollment?	◯ Yes<br>◯ No
Participant Age	__________________________________
Was the participant interviewed during this visit?	◯ Yes<br>◯ No
If no, why was the participant not interviewed?	◯ Moved to a different zone<br>◯ Wished to skip this visit<br>◯ Died<br>◯ Lost to follow up<br>◯ Withdrew permanently<br>◯ Not available today<br>◯ Other
If other, why was the participant not interviewed?	__________________________________
Phone number	__________________________________
Is this participant still in the same zone?	◯ Yes<br>◯ No

Occupation

Are you currently in school?<br><br>Je,ungali shuleni?<br><br>Gi esechegi bende pod idhi school?	◯ Yes<br>◯ No
What is the name of your school?<br><br>Shule yenu yaitwaje?	__________________________________

If yes: Is your school in Kisumu?

Shule yako ingali Kisumu?

Sikundu yudore Kisumu?

◯ Yes
◯ No

If yes: Is your school in Ukunda?

Shule yako ingali ukunda?

Sikundi yudore ukunda?

◯ Yes
◯ No

If no, where is your school?

Je, shule yako iko wapi?

Sikundi ni kanye?

INSTRUCTIONS FOR DATA COLLECTOR: Try to get as close to the exact location as possible by asking whatever would be needed for you to pinpoint the location on a map. For instance, if the participant gives the name of a town and you know the place, you can stop there. If they cannot give a town name, try to get the name of the area or region.

Neighborhood/estate ______________________________

City/town ______________________________

County ______________________________

Region ______________________________

Country (other than Kenya) ______________________________

Do you board (sleep) there?

Je wewe hulala humo?

Inindo ga kuro?

◯ Yes
◯ No

Do you have a job or do any work to earn money?

Gani ndio kazi yako muhimu?

En tich mane mitimo kaka yuto mari maduong?

◯ Yes
◯ No

Do you have a job or do any work to earn money?<br><br>Gani ndio kazi yako muhimu?<br><br>En tich mane mitimo kaka yuto mari maduong?	◯ Agricultural worker or farmer<br>◯ Artisan or craftsman<br>◯ Business Owner<br>◯ Butcher or slaughterhouse worker<br>◯ Clerical<br>◯ Cook<br>◯ Domestic service<br>◯ Driver<br>◯ Electrician<br>◯ Fisherman<br>◯ Gardener<br>◯ Government Worker<br>◯ Guard<br>◯ Healthcare worker<br>◯ Home Caregiver<br>◯ Hotel room keeping/service<br>◯ Manual Laborer<br>◯ Merchant or trader<br>◯ Military<br>◯ Office worker<br>◯ Police<br>◯ Researcher<br>◯ Salon worker<br>◯ Sells goods in a market or small shop<br>◯ Student<br>◯ Teacher<br>◯ Technical or managerial<br>◯ Tourism<br>◯ Traditional Healer<br>◯ Veterinarian<br>◯ Other
If "other" what is your primary job?<br><br>Kama nyingine, gani ndio kazi yako muhimu?<br><br>Kantie moro itiyo tich mane mar yuto ni pile kapile?	__________________________________
Do you have a second type of work to earn money?<br><br>Gani ndio kazi yako muhimu?<br><br>En tich mane mitimo kopogore gi mitimo ga pile?	◯ Yes<br>◯ No

Do you have a second type of work to earn money?

Gani ndio kazi yako muhimu?

En tich mane mitimo kopogore gi mitimo ga pile?

- ◯ Agricultural worker or farmer
- ◯ Artisan or craftsman
- ◯ Business Owner
- ◯ Butcher or slaughterhouse worker
- ◯ Clerical
- ◯ Cook
- ◯ Domestic service
- ◯ Driver
- ◯ Electrician
- ◯ Fisherman
- ◯ Gardener
- ◯ Government Worker
- ◯ Guard
- ◯ Healthcare worker
- ◯ Home Caregiver
- ◯ Hotel room keeping/service
- ◯ Manual Laborer
- ◯ Merchant or trader
- ◯ Military
- ◯ Office worker
- ◯ Police
- ◯ Researcher
- ◯ Salon worker
- ◯ Sells goods in a market or small shop
- ◯ Student
- ◯ Teacher
- ◯ Technical or managerial
- ◯ Tourism
- ◯ Traditional Healer
- ◯ Veterinarian
- ◯ Other

If "other" what is your secondary occupation?

Kama nyingine, gani ndio kazi yako ya pili?

Kantie moro tich mane mitimo ga tenge?

____

Education

What is your highest level of education?

Je, masomo yako ya juu ni gani?

Sombi mamalo en mane?

- ◯ No education
- ◯ Some primary school
- ◯ Completed primary school
- ◯ Some secondary school
- ◯ Completed secondary school
- ◯ College/university
- ◯ Technical college
- ◯ Other

If other, what is your highest level of education?

____

If under 18 years old:<br><br>What is your mother's highest level of education?<br><br>Kama uko chini ya miaka 18, je mamako mzazi alifika wapi kwa masoma?<br><br>Kain e bwo higa apar ga abor, somo mamalo mar mamani en mane?	◯ No education<br>◯ Some primary school<br>◯ Completed primary school<br>◯ Some secondary school<br>◯ Completed secondary school<br>◯ College/university<br>◯ Technical college<br>◯ Don't know<br>◯ Other
If other, what is your mother's highest level of education?	______________________________

Dengue and Chikungunya Knowledge Questions

Before you were enrolled to this study, had you heard of dengue?<br><br>Kabla ya kusajiliwa kwenye huu mradi, je uliwai sikia kuhusu dengue?<br><br>Ka pok idonjo e nonro ni, bende isega winjo tuo mar dengue?	◯ Yes<br>◯ No
If yes, where did you hear about it?<br><br>Kama ndio, ulisikia wapi?<br><br>Kaisewinjo, niwinje kanye?	☐ Family member or relative<br>☐ Friend<br>☐ Neighbor<br>☐ Healthcare worker<br>☐ Sign somewhere<br>☐ Newspaper<br>☐ Radio<br>☐ Another research project<br>☐ Other
Do you do anything to prevent getting dengue?<br><br>Kama ndio, je unafanya chochote kuuzuia?<br><br>Kantie,bende nitie gima itime mar geng'e?	◯ Yes<br>◯ No
If yes, what do you do to prevent dengue?<br><br>Kama ndio, nini?<br><br>Kanitie en ang'o?	____________________________________

Before you were introduced to this study, had you heard of chikungunya?<br><br>Kabla ya kusajiliwa kwenye huu mradi, je uliwai sikia kuhusu chikungunya?<br><br>Ka pok idonjo e nonro ni, bende isega winjo tuo mar chikungunya?	◯ Yes<br>◯ No
If yes, where did you hear about it?<br><br>Kama ndio, ulisikia wapi?<br><br>Kaisewinjo, niwinje kanye?	☐ Family member or relative<br>☐ Friend<br>☐ Neighbor<br>☐ Healthcare worker<br>☐ Sign somewhere<br>☐ Newspaper<br>☐ Radio<br>☐ Another research project<br>☐ Other
Do you do anything to prevent getting chikungunya?<br><br>Kama ndio, je unafanya chochote kuuzuia?<br><br>Kantie,bende nitie gima itime mar geng'e?	◯ Yes<br>◯ No
If yes, what do you do to prevent chikungunya?<br><br>Kama ndio, nini?<br><br>Kanitie en ang'o?	________________________

Moved

During the last 6 months, have you spent time living and sleeping outside your home?<br><br>Kwa muda wa miezi 6 iliopita, je umewai ishi na kulala mbali na boma lenu?<br><br>Kuom dweche auchie (6) mosekadho,bende isedak kata nindo oko mabor gi dalani?	◯ Yes<br>◯ No
If yes, where did you stay?<br><br>Kama ndio, uliishi wapi?<br><br>Ka isedak en kanye?<br><br>INSTRUCTIONS FOR DATA COLLECTOR: Try to get as close to the exact location as possible by asking whatever would be needed for you to pinpoint the location on a map. For instance, if the participant gives the name of a town and you know the place, you can stop there. If they cannot give a town name, try to get the name of the area or region.	

Neighborhood/estate	__________________
City/town	__________________
County/region	__________________
Country (if other than Kenya)	__________________
If yes, how long did you stay there?<br><br>Kama ndio, ulishi huko kwa muda gani?<br><br>Ka isedak,isebet kuro kuom ndalo marom nade?	◯ Less than 1 week<br>◯ 1 week - 1 month<br>◯ 1 month - 3 months<br>◯ 3-6 months<br>◯ Other
If "other," how long did you stay there?<br><br>Note to interviewer: Please feel free to add any comments regarding the participant's movements here	__________________________
Is there a second place you lived and slept during the last 6 months?<br><br>Kuna pahali pa pili ulishi na kulala kwa muda wa miezi 6 iliopita?<br><br>Bende nitiere kamoro ma ariyo misedak kata nindo oko mabor gi dalani?	◯ Yes<br>◯ No

If yes, where did you stay?

INSTRUCTIONS FOR DATA COLLECTOR: As above, try to get as close to the exact location as possible.

Neighborhood/estate	__________________
City/town	__________________
Country (if other than Kenya)	__________________
County/Region	__________________
If yes, how long did you stay there?	◯ Less than 1 week<br>◯ 1 week - 1 month<br>◯ 1 month - 3 months<br>◯ 3-6 months<br>◯ Other

If "other," how long did you stay there?<br><br>Note to interviewer: Please feel free to add any comments regarding the participant's movements here	____________________
Is there a third place you lived and slept during the last 6 months?<br><br>Kuna pahali pa tatu ulishi na kulala kwa muda wa miezi 6 iliopita?<br><br>Bende nitiere kamoro ma adek misedak kata nindo oko mabor gi dalani?	◯ Yes<br>◯ No
If yes, where did you stay?<br><br>INSTRUCTIONS FOR DATA COLLECTOR: As above, try to get as close to the exact location as possible.	
Neighborhood/estate	____________________
City/town	____________________
Country (if other than Kenya)	____________________
County/Region	____________________
If yes, how long did you stay there?	◯ Less than 1 week<br>◯ 1 week - 1 month<br>◯ 1 month - 3 months<br>◯ 3-6 months<br>◯ Other
If "other," how long did you stay there?<br><br>Note to interviewer: Please feel free to add any comments regarding the participant's movements here	____________________
Is there a fourth place you lived and slept during the last 6 months?<br><br>Kuna pahali pa nne ulishi na kulala kwa muda wa miezi 6 iliopita?<br><br>Bende nitiere kamoro ma ang'wen misedak kata nindo oko mabor gi dalani?	◯ Yes<br>◯ No
If yes, where did you stay?<br><br>INSTRUCTIONS FOR DATA COLLECTOR: As above, try to get as close to the exact location as possible.	

Neighborhood/estate	__________________________
City/town	__________________________
Country (if other than Kenya)	__________________________
County/Region	__________________________
If yes, how long did you stay there?	◯ Less than 1 week<br>◯ 1 week - 1 month<br>◯ 1 month - 3 months<br>◯ 3-6 months<br>◯ Other
If "other," how long did you stay there?<br><br>Note to interviewer: Please feel free to add any comments regarding the participant's movements here	________________________________
Is there a fifth place you lived and slept during the last 6 months?<br><br>Kuna pahali pa tano ulishi na kulala kwa muda wa miezi 6 iliopita?<br><br>Bende nitiere kamoro ma abich misedak kata nindo oko mabor gi dalani?	◯ Yes<br>◯ No
If yes, where did you stay?<br><br>INSTRUCTIONS FOR DATA COLLECTOR: As above, try to get as close to the exact location as possible.	
Neighborhood/estate	__________________________
City/town	__________________________
Country (if other than Kenya)	__________________________
County/Region	__________________________
If yes, how long did you stay there?	◯ Less than 1 week<br>◯ 1 week - 1 month<br>◯ 1 month - 3 months<br>◯ 3-6 months<br>◯ Other

If "other," how long did you stay there?

Note to interviewer: Please feel free to add any comments regarding the participant's movements here ______________________

Mosquito Exposure

Does your household have bednets?

Je jamii yako muko na neti ya mbu?

Bende odi nitie gi net mar suna?

- ◯ Yes
- ◯ No

How many bednets do you have? ______________________

Je, muko na neti ngapi za mbu?

Un gi nede adi mag suna?

Did you sleep under a mosquito net last night?

Je, ulilala ndani ya neti ya mbu usiku uliopita?

Bende ni nindo ei net mar suna nyoro gotieno?

- ◯ Yes
- ◯ No

How often do you sleep under a mosquito net?

Ni mara ngapi unalala ndani ya neti ya mbu?

Inindo ga e buo net kuom kinde marom nade?

- ◯ Always
- ◯ Sometimes
- ◯ Rarely
- ◯ Never

When is the last time your house got a new mosquito net?

Ni lini mara ya mwisho mulipata neti mpya ya mbu?

Nuyodo net manyien kar ang'o?

- ◯ Less than 1 month ago
- ◯ 1-6 months ago
- ◯ 7-12 months ago
- ◯ 1-5 years ago
- ◯ more than 5 years ago

When you got your nets, were any of them treated with insecticides to repel or kill mosquitoes before you bought them?

Ulipopata neti zako,kulikuwa na ziliyotibiwa na dawa ya kuzuia wadudu ama kuua mbu kabla ya kuzinunua?

Kaniyudo net,bende nenitie moro kuomgi mane othiethi gi yath mar geng'o suna kata negogi kapok ninyiewogi?

- ◯ Yes
- ◯ No
- ◯ Don't know

Have any of your bednets been treated with insecticides after they were purchased?<br><br>Je, kuna neti ya mbu yeyote imewai wekwa dawa ya wadudu baada ya kununuliwa?<br><br>Nyaka nunyiew nede gi bende nite mosethiedhi gi yath kute?	◯ Yes<br>◯ No<br>◯ Don't know
If yes, how many months ago were they treated with insecticide?<br><br>Kwa mda wa miezi mingapi zilizopita zilitibiwa na dawa ya kuzuia wadudu?<br><br>Kuom kinde marom nade mane ithiethо gi gi yath geng'o suna?<br><br>INSTRUCTIONS FOR DATA COLLECTOR: Write in a number, or else type in 999 if the answer is "I don't know"	______________________________<br>(Please enter 999 if the respondent doesn't know how long it takes to get water)
In the past two weeks, have you been bitten by mosquitoes?<br><br>Kwa muda wa juma mbili zilizopita, je umewai umwa na mbu?<br><br>Ei kinde mar jumbe ariyo mokadho bende suna osekayi?	◯ Yes<br>◯ No<br>◯ Don't Know
If yes, when did the mosquitoes bother you?<br><br>Kama ndio, zilikuuma wakati gani?<br><br>To ka osekayi nokayi seche mage?	☐ Day<br>☐ Night<br>☐ Don't know
Approximately how often are you bitten by mosquitoes?<br><br>Kwa kadri, unaumwa na mbu mara ngapi?<br><br>Kinyalo pimo suna kayi ga marom nade?	◯ Daily<br>◯ Weekly<br>◯ Monthly<br>◯ Yearly<br>◯ Don't Know

Mosquito Control

In the past two weeks...
Kwa juma mbili zilizopita...
Kuom jumbe ariyo mokadho...

	Yes	No	Don't Know
Have you used mosquito repellent? / Je, umejipaka mafuta ya kufukuza mbu? / Bende isewirori gi moo mariembo suna?	◯	◯	◯
Have you used mosquito coils in/around your house? / Je, umetumia dawa ya kuchoma ya kufukuza mbu ndani au karibu na nyumba yako? / Bende isetiyo gi yath miwang'o mar riembo suna ei ot kata aluora mara ot?	◯	◯	◯
Have you used insecticide in/around your house? / Je, umetumia dawa ya wadudu ndani au karibu na nyumba yako? / Bende isetiyo gi yath kute iot kata aluora mar ot?	◯	◯	◯
Has anyone come into your house to spray the interior walls against mosquitoes? / Je, kuna mtu yeyote aliyepuliza dawa ya mbu kwa kuta za ndani ya nyumba yako? / Bende ng'ato ang'ata osebiro go yath suna e kor ot mar ii odi?	◯	◯	◯

Latest Fever Questions

Have you had a fever in the last six months?

Je, umekuwa na joto jingi mwilini kwa muda wa miezi 6 iliopita?

Bende isebet gi liet mar del kuom kinde mar dweche 6 mokadho?

◯ Yes
◯ No

If yes, how long did the symptoms for your latest fever last?

Kama ndio, hizi dalili zilikuwa kwa muda gani?

Ka isebet go, ranyisi gi nibet go kuom kinde kata ndalo marom nade?

FOR THE INTERVIEWER: Please enter how long the participant's fever lasted. Not all fields need to be filled out. For example, if the participant says "2 days," just put "2" in the 'days' field and leave the other fields blank. If "3 months," put "3" in the 'months' field

Days ______________________

Weeks ______________________

Months ______________________

If yes, did you seek medical care for your most recent fever?

Kama ndio, ulitafuta huduma ya matibabu kwa joto jingi mwilini uliokuwa nayo hivi karibuni?

Kaisebet go,bende nimanyo yor thieth ne liet mar del manyocha in godo machiegni ni?

◯ Yes
◯ No
◯ Don't Know

Did you see a Clinical Officer FROM THIS STUDY while you were sick?

Kama ndio, afisa wa matibabu wa utafiti huu alikuja kukuona ulipokuwa na joto jingi mwilini?

To kanitie, bende laktar mochung ne thieth mar nonroni noneni kane in gi liet mar del?

◯ Yes
◯ No

If yes, where did you see the clinical officer?

Kama ndio, mulionana wapi na afisa wa matibabu?

To ka ee,nineno laktar mochung ne thieth kanye?

◯ At home
◯ At healthcare center
◯ Other

If "Other" where did you see the clinical officer? ______________________

If you did not seek care form a clinical officer from this study, what was the source of this care?<br><br>Kama ndio, ulipata wapi haya matibu?<br><br>To kane imanyo, niyudo thieth kanye?	☐ Clinic or hospital<br>☐ Community health worker outside a clinic or hospital<br>☐ Pharmacy or chemist<br>☐ Care from a family member or acquaintance NOT trained as healthcare worker<br>☐ Traditional healer<br>☐ Other
If other, what other type of medical care did you receive?	__________________________________
If a clinic or hospital, which clinic or hospital did you go to?<br><br>Ulienda kwa hospitali gani?<br><br>Nidhi osiptal mane?	☐ Obama Childrens Hospital<br>☐ Likoni Health Center<br>☐ Jaramogi Odinga Referral Hospital<br>☐ Ukunda Medical Center<br>☐ Kisumu Hospital<br>☐ Msambweni County Referral Hospital<br>☐ Migosi Subcounty Referral Hospital<br>☐ Kwale Subcounty Hospital<br>☐ Other
If other, specify where the participant sought medical care.	__________________________________

Symptoms during most recent fever

Wakati ulikuwa na joto jingi mwilini hivi karibuni, ni ishara gani zingine ulikuwa nazo?

E seche mane in gi liet mar del nyocha machigni, ranyisi mane kendo mane in go?

Instructions for clinical officer: ask the participant about EACH specific organ system listed below. If they say they had a problem with that organ system, check the box and then you will be prompted to specify exactly what the problem was.

	Yes	No	Unsure/Don't Know
PROBLEMS WITH THE HEAD, BRAIN, or NECK / Shida za kichwa, ubongo ama shingo / In gi rem mar wich,obuongo kata ng'uti	○	○	○
Confusion	○	○	○
Dizziness	○	○	○
Headache	○	○	○
Neck swelling	○	○	○
Seizures	○	○	○
Stiff neck	○	○	○

PROBLEMS WITH EYES, EARS, NOSE, or THROAT / Shida za macho, maskia, mapua ama koo / In gi rem mar wang'I it,umi kata duondi	◯	◯	◯
Eye discharge	◯	◯	◯
Eye pain	◯	◯	◯
Eye redness	◯	◯	◯
Yellow eyes	◯	◯	◯
Blurry vision	◯	◯	◯
Ear discharge	◯	◯	◯
Ear pain	◯	◯	◯
Runny nose	◯	◯	◯
Sore throat	◯	◯	◯
PROBLEMS WITH CHEST OR BREATHING / Shida za kupumua ama kifua / In gi rem mar yueyo kata kori	◯	◯	◯
Chest Pain	◯	◯	◯
Cough	◯	◯	◯
Difficulty breathing	◯	◯	◯
STOMACH PROBLEMS / Shida za tumbo / In gi ich maremo	◯	◯	◯
Abdominal Pain	◯	◯	◯
Constipation	◯	◯	◯
Diarrhea	◯	◯	◯
Nausea or Vomiting	◯	◯	◯
PROBLEMS WITH MUSCLES, JOINTS, OR LIMBS / Shida za kuuma na misuli ama viungo / In gi rem mar ondhundho kata fuondni ni	◯	◯	◯
Joint pain	◯	◯	◯
Joint stiffness	◯	◯	◯
Joint swelling	◯	◯	◯
Muscle pain	◯	◯	◯
Back pain	◯	◯	◯
Flank pain	◯	◯	◯
Numbness in part of the body	◯	◯	◯
Weakness in part of the body	◯	◯	◯

PROBLEMS WITH SKIN OR BLOOD / Shida za ngozi ama damu / Rem mar pien del gi remo	○	○	○
Abnormal bleeding	○	○	○
Itching	○	○	○
Rashes	○	○	○
Sores	○	○	○
Dark urine	○	○	○
Other	○	○	○

During your most recent illness with fever, which other symptoms did you experience?

____

Did you receive a diagnosis for your illness?

○ Yes
○ No

If you received a diagnosis for your illness, what was the diagnosis?

☐ Unclear diagnosis
☐ Anemia
☐ Bacterial infection
☐ Chikungunya
☐ Dengue
☐ Ear infection
☐ Eye infection
☐ Gastritis
☐ Gastroenteritis
☐ HIV/AIDS
☐ Intestinal parasite(s)
☐ Malaria
☐ Meningitis
☐ Peptic ulcer
☐ Pneumonia
☐ Schistosomiasis
☐ Sickle cell crisis
☐ Skin infection
☐ Tonsilitis/Pharyngitis
☐ Tuberculosis
☐ Typhoid
☐ Upper respiratory tract infection ('cold')
☐ Lower respiratory tract infection ('pneumonia')
☐ Urinary Tract Infection
☐ Other

If other, what other diagnosis did the participant receive for their illness?

____

General Health

Have you been diagnosed with any medical conditions?

○ Yes
○ No

If yes, which ones?	☐ None<br>☐ Asthma<br>☐ Cancer<br>☐ Cerebral palsy<br>☐ Diabetes<br>☐ Epilepsy<br>☐ Heart disease<br>☐ HIV<br>☐ Hypertension<br>☐ Mental illness<br>☐ Peptic ulcer<br>☐ Sickle cell disease<br>☐ Stroke<br>☐ Tuberculosis<br>☐ Other<br>☐ Refuse
Which other medical conditions have you been diagnosed with?	__________________________
For children under 5, was the child full term or preterm?<br><br>Kwa watoto wa umri chni ya miaka 5: huyu mtoto alizaliwa kwa wakati wake au kabla ya wakati wake?<br><br>Ne nyithindo mane ebuo higa 5: bende nyathi nonyuol kandalo ne oromo koso kapodi?	◯ Full term<br>◯ Preterm<br>◯ Do not know
For children under 5, was the child breast fed as a child?<br><br>Kwa watoto wa umri chni ya miaka 5: huyu mtoto alinyonyeshwa?<br><br>Ne nyithindo mantie e buo higni 5: bende nyathino nodhodhi?	◯ Yes<br>◯ No<br>◯ Don't Know
If yes, how many months was the participant breast fed?<br><br>Kama ndio, huyu mtoto alinyonyeshwa kwa miezi ngapi?<br><br>Kane odhoth nodhodhe kuom dweche marom nade?	◯ 1 - 6 month<br>◯ 7 - 12 months<br>◯ 13 - 24 months<br>◯ Greater than 24 months<br>◯ Do not know
Are you currently pregnant?<br><br>Je, kwa sasa we ni mja msito?<br><br>Gi seche gi iyach?	◯ Yes<br>◯ No

About how many months pregnant are you?

Kama ndio, takriban miezi mingapi ya uja usito?

Ka iyach, en dweche adi kama?

____

We notice that there may be rift valley fever virus (RVFV) in this area. I am now going to ask you some questions to assess your risk of disease exposure

In which County were were you born?

____

How long have you lived in this (current) house?

____
(Indicate number and specify if it is months or years)

How long have you lived in Kisumu?

____
(Indicate number and specify if it is months or years)

How long have you lived in Ukunda?

____
(Indicate number and specify if it is months or years)

Which of these animals do you own?

- ☐ Cows for milk
- ☐ Cows for beef
- ☐ Goats
- ☐ Sheep
- ☐ Camels
- ☐ Pigs
- ☐ Poultry
- ☐ Dogs
- ☐ Cats
- ☐ No animals

Number of cows for milk

____

Number of cows for beef

____

Number of goats

____

Number of sheep

____

Number of camels

____

Number of pigs

____

Number of poultry	________________________________
Number of dogs	________________________________
Number of cats	________________________________
Where do you keep these animals?	◯ Around personal home<br>◯ Offsite<br>◯ Other
Where else do you keep these animals?	________________________________
Do you have ruminants (cows, sheep, goats)?	◯ Yes<br>◯ No
If yes, who grazes them?	☐ Myself<br>☐ Son<br>☐ Daughter<br>☐ Other family member<br>☐ Hired laborer<br>☐ Other
If other, who grazes them?	________________________________
If yes, who milks the ruminants?	☐ Myself<br>☐ Son<br>☐ Daughter<br>☐ Other family member<br>☐ Hired laborer<br>☐ Other
If other, who milks the ruminants?	________________________________
Within the past month, have you see any of the following animals around your home that do not belong to you?	☐ Cows for milk<br>☐ Cows for beef<br>☐ Goats<br>☐ Sheep<br>☐ Camels<br>☐ Pigs<br>☐ Poultry<br>☐ Dogs<br>☐ Cats<br>☐ Rodents<br>☐ Bats<br>☐ Buffaloes<br>☐ Antelope<br>☐ None<br>(Read off each animal and tick all that apply)
Do any animals enter your home?	◯ Yes<br>◯ No<br>◯ N/A

If yes, which animals?	☐ Cows for milk<br>☐ Cows for beef<br>☐ Goats<br>☐ Sheep<br>☐ Camels<br>☐ Pigs<br>☐ Poultry<br>☐ Dogs<br>☐ Cats<br>☐ Rodents<br>☐ Bats<br>☐ Buffaloes<br>☐ Antelope<br>(Tick all that apply)
Do you provide care for any animals including ones that do not belong to you?	◯ Yes<br>◯ No
What care activities do you provide?	☐ Milking<br>☐ Cleaning waste<br>☐ Feeding<br>☐ Assisting births<br>☐ Providing medical care<br>☐ Herding<br>☐ Other
What other care activities do you provide?	__________________________________
Have you assisted an animal giving birth in the past 1 month?	◯ Yes<br>◯ No<br>◯ N/A
If yes, what animal?	☐ Cows for milk<br>☐ Cows for beef<br>☐ Goats<br>☐ Sheep<br>☐ Camels<br>☐ Pigs<br>☐ Poultry<br>☐ Dogs<br>☐ Cats<br>☐ Rodents<br>☐ Bats<br>☐ Buffaloes<br>☐ Antelope
Have you ever participated in slaughtering an animal ?	◯ Yes<br>◯ No
If yes, what animal?	☐ Chicken<br>☐ Goat<br>☐ Sheep<br>☐ Cattle<br>☐ Camel<br>☐ Rabbit<br>☐ Other
What other animal did you participate in slaughtering?	__________________________________

Do you drink raw milk?	◯ Yes<br>◯ No
How often do you drink raw milk?	◯ Daily<br>◯ Two times per week<br>◯ Weekly<br>◯ Two times per month<br>◯ Monthly<br>◯ Yearly
Where do you get this raw milk from?	☐ Personal animal<br>☐ Direct from neighbors<br>☐ Supermarket<br>☐ Milk vendors<br>☐ Small shops
How do you prepare raw milk before consuming?	☐ Nothing, consume raw<br>☐ Boiling<br>☐ Fermentation<br>☐ Other
How else do you prepare milk before consuming?	__________________________________
How often do you prepare beef for cooking?	◯ Daily<br>◯ Two times per week<br>◯ Weekly<br>◯ Two times per month<br>◯ Monthly<br>◯ Yearly<br>◯ Never
Where do you get this beef?	☐ Personal animals<br>☐ Market (live)<br>☐ Market (cuts)<br>☐ Direct purchases from neighbors
How often do you prepare goat meat for cooking?	◯ Daily<br>◯ Two times per week<br>◯ Weekly<br>◯ Two times per month<br>◯ Monthly<br>◯ Yearly<br>◯ Never
Where do you get this goat meat?	☐ Personal animals<br>☐ Market (live)<br>☐ Market (cuts)<br>☐ Direct purchases from neighbors
How often do you prepare sheep meat for cooking?	◯ Daily<br>◯ Two times per week<br>◯ Weekly<br>◯ Two times per month<br>◯ Monthly<br>◯ Yearly<br>◯ Never

Where do you get this sheep meat?	☐ Personal animals<br>☐ Market (live)<br>☐ Market (cuts)<br>☐ Direct purchases from neighbors
How often do you butcher live animals?	◯ Daily<br>◯ Two times per week<br>◯ Weekly<br>◯ Two times per month<br>◯ Monthly<br>◯ Yearly<br>◯ Never
In the last month, what animals have you butchered?	☐ Chicken<br>☐ Goat<br>☐ Sheep<br>☐ Cattle<br>☐ Pig<br>☐ None
What protective clothing do you wear when you butcher an animal?	☐ Gloves<br>☐ Mask<br>☐ Boots<br>☐ Eye protection<br>☐ Aprons<br>☐ White laboratory coat<br>☐ None<br>☐ Other<br>☐ Do not know<br>(Tick all that apply)
What other protective clothing do you wear when you butcher an animal?	__________________________________
Do you use animal blood for anything?	__________________________________
Do monkeys enter your compound?	◯ Yes<br>◯ No<br>◯ No monkeys in my area
Do people in this area slaughter any wild animals?	◯ Yes<br>◯ No
Which wild animals do people slaughter in this area?	☐ Monkeys<br>☐ Wild hog<br>☐ Buffalos<br>☐ Antelopes<br>☐ Rodents<br>☐ None<br>☐ Other
What other wild animals?	__________________________________

Visit screening questions

These questions will help determine whether this visit will also be a 'sick visit'

Do you have fever today?	◯ Yes<br>◯ No
Have you had fever in the last three days?	◯ Yes<br>◯ No
Participant temperature (Celsius)	__________________________
What was the source of the temperature reading above?	◯ Axillary<br>◯ Oral<br>◯ Tympanic

This participant is currently febrile (Temperature >=38 C), or has reported that they were febrile in the past three days.

Please refer the participant to the Clinical officer.

The participant is currently afebrile and has reported that they have been afebrile for the past three days.

End the survey.
