# Supplementary material for: Urban risk factors for human Rift Valley fever virus exposure in Kenya
Source: PLOS Glob Public Health. 2022 Jul 14;2(7):e0000505. doi: 10.1371/journal.pgph.0000505 (PMC10021321; doi:10.1371/journal.pgph.0000505)
Supplement: Attachment — Submitted filename: Response to reviewers.docx [file pgph.0000505.s003.docx]

Dear PLOS Global Public Health journal editor,

Thank you for the insightful responses to my manuscript submission: Urban risk factors for human Rift Valley fever virus exposure in Kenya. I have revised the manuscript and my specific responses to comments is included below. In my attached manuscript with track changes, I have highlighted yellow the specific changes that I completed.

As per the journal requirements:

1. I have provided a copy of the PLOS questionnaire on inclusivity in global health research.
2. My co-authors have been notified. You should have acceptance now from Francis Mutuku, Said Malumbo, Eleonora Migliore, Eduardo Fabre, Izabela Rezende, and Karren Shaita. I have attempted extensively to contact them and if they do not respond by the end of the week, I will be forced to remove them as authors
3. I have updated my financial disclosures statement to include the funders had no roles in my study.
4. My map has been updated to only include open-source data and a link to the base map that I used to create the image.
5. All figures have been uploaded as tiff images

Reviewer 1 and 2 comments have been addressed in full and my responses are in italics.

Reviewer 1 provided comments in an attached file. My responses and changes are enumerated:

1. Comment on p values vs OR in the abstract and inconsistency in reporting significant values in abstract.
   a. *Added the ORs, CI 95% and p values from the multivariate modeling as suggested in the abstract*
   b. *All p values have leading zero and are italicized. All mentions of 95% confidence intervals have been changed to CI 95% throughout the manuscript for consistency*
2. *Line 235: Edited sentence as suggested “Focus was directed on significance of predictors based on the Wald test p-value. The Akaike Information Criterion (AIC) was recorded to monitor model quality as insignificant variables were removed.”*
3. *Line 259: Added p values to statements about older males having higher seroprevalence and p value to compare age groups in Kisumu to make point*
4. *Line 268: Eliminated use of the term “co-exposure” and changed to “had antibodies indicating prior exposure to at least one other arbovirus”*
5. *Line 274: added “at any time over their lifetime.” to the end of the sentence*
6. *Line 297: Changed multivariate to “multivariable”*
7. *Table 2: I have updated the p-values reported to be from the logistic regressions and highlighted this in the title as suggested rather than the p values from Fischers exact test*
8. *Line 303: Correlation test has been updated to be Pearsons correlation test (rather than Spearmans). I have formatted the results of the Pearsons test to APA format and changed this in the methods. My r values only changed slightly and did not affect the initial model inputs Thank you for catching this.*
9. *Table 4: I have clarified the title that I am only reporting the odds ratios here. I did not complete a multivariate analysis on this matched case control study as there are a limited number of outcomes and the variables, we matched on were inherently controlled for. I am only reporting the simple bivariate analysis here in this table.*
10. *Line 444: Note about ending discussion on a negative note: I moved my limitations paragraph up so that the spirit of the discussion is more positive at the end*
11. *Line: 453: added vector bites to animal product consumption*

Reviewer #2 Specific comments were as followed and my response is italicized:

1. Line 81: Yes, many mammal species tested do appear to replicate RVFV. However, does RVFV replicate in any avian species? You might want to change “animals,” which includes birds, to “mammals.”

*Changed animals to mammals*

2. Line 101: Yes, I know that Kisumu and Ukunda are indicted in Figure 1, but why not modify this sentence to, “…two urban sites in Kenya, Kisumu and Ukunda, from December…?”

*Changed as recommended*

3. Line 132: Use of the word, “breed.” I know that breed is commonly used to refer to mosquito larval habitat, but breed has sexual implication and mosquitoes do not breed in water, their larvae develop in water. Shouldn’t this be changed to, “… where mosquitoes mostly develop (28)?”

*I have changed this sentence to “Some sections of the city are well-developed (planned) and maintained as opposed to others which are poorly drained (unplanned), where water accumulates and attracts breeding mosquitoes (28).”*

4. Line 333, Table 4: As with comments xx and xx below, I have some questions about the numbers in Table 4. There appears to be 15 “cases’ for most of the variables, i.e., milk source, purchase from vendors, community animals, and health. Why are there data from only 14 cases about purchasing milk from dairy cow owners or for handling ruminant blood, or from 21 cases for adding spoiled milk to cooked vegetables? Note, if the 18 cases adding spoiled milk to their vegetables was really 12 cases (to have 15 total cases), then wouldn’t the OR be over 1.5? Minor, but capitalization appears to be at random, i.e., Milk is capitalized in some but not other variables. Similar comment for ruminant.

*Corrected capitalization of ruminant and milk throughout and also blurry vision. Default to first letter capitalized only. I have chosen to omit the line about adding milk to cooked vegetables. The way this data was collected was as a household level exposure and is confusing to present in the analysis. At this point, I have also learned more about this practice and see that the milk is indeed boiled with the vegetables and is unlikely to be a source of undercooked milk consumption.*

5. Line 343: Here it states that one person was involved in slaughtering animals, yet in table 2, it states than 0/1,201 of the participants slaughtered animals. Which is it?

*I have clarified table 2. This question was meant to capture anyone that had recently slaughtered (in the past 1 month) and the second time the question is asked is if anyone had ever slaughtered in their lifetime*

6. Lines 368-369: On line 368 it states that 97/105 of the participants reported willingness to take the vaccine (i.e., 8 unwilling), but on the next line, it states that of the nine seropositive participants that would refuse… How could there be nine refusers? More importantly, if the second sentence is correct, then the refusal rate was 9/21 for the positive cases and 0/84 for the negative controls. That is amazingly significant, so much so that I think the sentence is wrong.

*Thank you for detecting this error. There were eight refusers and one of them was seropositive. The sentence now reads “Of the eight participants that would refuse, one participant was seropositive for RVFV and they reasoned that they would not accept the vaccination unless they had the disease”*

7. Lines 373-375: Is there a RVFV vaccine approved for human use in Kenya? If not, how could the nine participants have previously declined a vaccination for their child? Minor, but “nine seropositive” on lines 369 and “nine participants” on line 373 should be “9 seropositive” and “9 participants,” respectively.

*I have not corrected the “nine” to 9 because I have standardized throughout spelling out numbers less than 10. There is no RVFV vaccination for humans approved for general population. This question was regarding overall vaccination acceptance. I have updated this sentence to be more clear: “Of the 9 participants that said they had previously declined their child vaccination for another infectious disease, four had RVFV exposure”*

Minor comments:

8. Lines 111-112: As “months” are a unit of measurement, shouldn’t this be “6 months after…?” See also line 141 where “two kilometers south…” should be 2 kilometers south…” or “2 km south…” Throughout the manuscript, I’m not certain if it should be “kilometers” or “km” and “meters” or “m.”

*I have updated the consistency of this throughout and opted to use kilometers (km) before using the abbreviations. I have also spelled out all numbers less than 10 throughout unless it is in a table or part of an odds ratio*

9. Line 161: Minor, but “RVFV exposed children” should be “RVFV-exposed children.”

10. Line 253, Figure 2: I am just curious as to why the number positive for RVFV IgG is formatted differently in the Community Cohort, “57 positive…(1.6%),” and the Available for Follow-Up, “(RVFV anti-RVFV IgG+ n=35).” Why not use the same format and use “35 positive anti-RVFV IgG (1.6%)?” Note, the positivity rate remained at 1.6%.

11. Line 263, Table 1: Why is the “0.02” for gender in bold? Yes, it indicates that gender is a significant predictor of the presence of RVFV antibodies, so I like it being highlighted, but what about the other significant ones? Either highlight all or none of them.

*Corrected # 9 through 11 as recommended.*

12. Line 279, Table 2: Are the numbers for consumption of raw milk correct? How could there be 79 total when there were 25 each in Kisumu and Ukunda? Similarly, if there were only 1,201 people, how could there be 16 who assisted a livestock birth and 1,192 who did not (16 + 1,192 = 1,208)?

*Corrected, this should have been 1,185 who did not assist livestock birth*

13. Line 334: Shouldn’t “9 times more…” be “nine times more…?” Similar comment for line 336, and why is “Seropositive” capitalized?

*Corrected both*

14. Line 357: Shouldn’t “twelve participants…” be “12 participants…?” also, line 350. Shouldn’t (5 cases) be (five cases) and line 269 where 9 seropositive should be nine seropositive?

*I have corrected this throughout t2o spell out numbers less than 10. Twenty was spelled out because it started a sentence but I have modified this to “There were 20 (five cases) participants who purchased milk from dairy owners directly and two justified their decision”*

12. Line 395-396: Why write out “animal sourced foods” when “ASFs” has been established and used several times?

*Indeed, corrected*

16. Lines 404-405: Was the seroprevalence, about 2%, in Kisumu significantly greater than that in Ukunda, about 1%? Yes, any seroprevalence is too high, but without giving any numbers in the discussion, simply stating, “The high seroprevalence in children aged 5-17 years in Kisumu is concerning” is a bit misleading.

*I’ve added the % from the table and provided a p-value. “The higher seroprevalence in children aged 5-17 (2.1% compared to 1.6% in adults) in Kisumu is concerning and we did not find any direct epidemiological links to explain this finding”*

17. Lines 412-413: Comments about poultry. First of all, as sort of mentioned, I do not believe that RVFV produces a viremia in poultry, and as such, they would not be involved in RVFV transmission. Rather than serve as an attractant for potential vectors, they are likely to reduce RVFV transmission, sort of like cattle and malaria, by drawing vectors away from other mammals that could produce a viremia and expand RVFV transmission.

*Correct, RVFV only causes viremia in goats, not poultry. Since poultry was not significant in multivariate analysis, I have omitted this part from the statement. There is no proven link of poultry presence and aedes mosquito reduction (the zooprophylaxis concept) because the aedes like the breed in poultry water dishes. This is indeed true for malaria though. I have made this clearer in my discussion line 415-418*

18. Lines 422-423: Here the authors stated, “in the urban setting, where handling of raw meat and milk is common.” As far as I can tell, theis is the first mention of “raw meat” in the manuscript. Was there any difference in seroprevalence by consumption of raw meat? Second, according to Table 2, overall, <10% consumed raw milk. Is this really a “common” behavior?

*I am refereeing to handling not consuming. I have also changed meat and milk again to ASFs; the sentence now reads “Vaccinating humans for RVFV in urban populations will be challenging without fully understanding who is at higher risk especially in the urban setting, where handling of ASFs is common and daily vector exposure is nearly unanimous.”*

19. Line 437: As the authors had just mentioned, “anti-RVFV antibodies are thought to be lifelong.” Thus, the fact that 80% of the RVFV exposed individuals had not had “recent” travel is misleading. Yes, the statement is meaningful, but I think that they need to qualify it better that “recent” travel may not have been the source.

*I’ve clarified this to reduce the chance of being misleading. “We collected information on travel outside of the urban setting to control for this possibility and 80% of RVFV exposed individuals had no recent outside travel (Table 1) which could indicate urban transmission potential, although we cannot collect lifetime travel information, and travel could have indeed been the source of infection for our urban participants”*

20. References: These need to be formatted properly.
a. Only the first word and proper nouns in a reference title should be capitalized. See reference 2. Also, in reference 7, “fever” in Rift Valley fever should not be capitalized and in reference 12, “yellow” should not be capitalized as they are not proper nouns.
b. All proper nouns need to be capitalized. “Rift Valley fever” appears as “rift valley fever” in numerous references. See references 5, 6, 8, and others.
c. I believe that there should be a “space” after the “colon” after the volume number and before the page number.
d. I believe that all pages should be indicated, i.e., in reference 2, “PLoS Negl Trop Dis. 2014;8(9):17–9” should have been, “PLoS Negl Trop Dis. 2014;8(9): 17–19.” These need to be fixed for many of the references.
e. Shouldn’t the journal for reference 28 be “Parasit Vectors” instead of “Parasites and Vectors?”

*Corrected as recommended*

Thank you kindly and I look forward to further correspondence regarding this manuscript.

Keli Gerken, DVM, MRCVS
